# Metagenome-Assembled Genomes Support the Proposal of *Candidatus Flavobacterium genomatis* from the Northeast Black Soil Ecosystem

**DOI:** 10.3390/microorganisms14061292

**Published:** 2026-06-08

**Authors:** Xiaoyue Zhang, Caiyu Lu, Luotian Lu, Liqiang Meng, Yalong Liu, Bin Ma

**Affiliations:** 1 State Key Laboratory of Soil Pollution Control and Safety, Zhejiang University, Hangzhou 310058, China; 22414158@zju.edu.cn (X.Z.); ltlu@zju.edu.cn (L.L.); 2 Zhejiang Provincial Key Laboratory of Agricultural, Resources and Environment, College of Environmental and Resource Sciences, Zhejiang University, Hangzhou 310058, China; 3 ZJU-Hangzhou Global Scientific and Technological Innovation Center, Zhejiang University, Hangzhou 311215, China; 4 Institute of Microbiology, Heilongjiang Academy of Sciences, 68 Zhaolin Street, Harbin 150010, China; mengliqiang83420@163.com; 5 College of Land and Environment, Shenyang Agricultural University, Shenyang 110866, China; yalongliu@aliyun.com

**Keywords:** *Flavobacterium*, metagenome-assembled genomes (MAGs), Global Mollisols Genomic Atlas (GMGA), ICNP

## Abstract

Soils are critical microbial habitats that support terrestrial ecosystem functioning and harbor numerous uncultured and functionally uncharacterized microbial groups. The black soil region in northeast China is a key agricultural ecosystem globally, yet the classification and functional understanding of its crucial microbial groups remain underexplored. In this study, we identified three high-completeness metagenome-assembled genomes (MAGs) from the Global Mollisols Genomic Atlas (GMGA). Phylogenetic and comparative genomic analyses identified these genomes as representing a novel evolutionary branch within the genus *Flavobacterium*, classified under the phylum *Bacteroidota*. Their novel taxonomic position is further supported by average nucleotide identity (ANI) and average amino acid identity (AAI) thresholds, demonstrating significant divergence from all known reference genomes. Functional annotation indicated that this species possesses strong plant polysaccharide degradation potential and a chemoheterotrophic lifestyle, together with environmental stress tolerance and a specialized nitrogen metabolic network adapted to agricultural inputs, thereby conferring a metabolic advantage in black soil environments characterized by high organic matter input and marked seasonal fluctuations. In addition, global distribution analysis showed that this lineage is widely distributed across diverse ecosystems and is significantly enriched in soil habitats, particularly in environments with fluctuating carbon sources and high organic matter inputs. The new species is most abundant in temperate soils, with the northeast black soil region of China emerging as a key hotspot. Based on these findings, and because no pure culture is currently available, we propose *Candidatus Flavobacterium genomatis* based on genome-resolved metagenomic evidence and in alignment with the International Code of Nomenclature of Prokaryotes rules for uncultivated prokaryotes. Our results expand the known species diversity of the genus *Flavobacterium* and suggest potential ecological roles of uncultured black-soil microbes in carbon and nitrogen cycling, including possible involvement in N_2_O reduction under suitable environmental conditions.

## 1. Introduction

Soil is one of the most biologically diverse habitats on Earth, harboring a wealth of uncultured microbial groups that are integral to global biogeochemical cycles and ecosystem functioning [1,2,3]. The black soil region in northeastern China, a globally vital agricultural zone, is characterized by its high organic matter content, deep humus layer, and severe seasonal freeze–thaw cycles [4]. These unique physicochemical properties make it a dynamic hotspot for carbon and nitrogen cycling. Black soils support robust microbial communities that drive the decomposition of recalcitrant organic matter, nutrient mineralization, and element cycling, thereby maintaining soil health and agricultural productivity [5,6]. Despite significant advances in metagenomics and high-throughput sequencing, the vast majority of soil microbes remain uncultured. Consequently, many microbial lineages in black soils still lack well-defined taxonomic frameworks and functional characterizations, representing a significant portion of “microbial dark matter” [7,8].

Within these soil microbial communities, the phylum *Bacteroidota* plays a pivotal role in organic matter degradation, particularly in breaking down complex, high-molecular-weight biopolymers like plant-derived polysaccharides. *Bacteroidota* is known for its ability to degrade complex organic compounds, making it a crucial player in the terrestrial carbon cycle [9,10]. Members of *Bacteroidota* are ubiquitous in environments rich in organic matter, such as soil and water, where they act as primary decomposers, converting recalcitrant carbon sources into simpler forms that are more readily available to other microorganisms [9]. Given the continuous influx of plant litter and agricultural residues in black soils, *Bacteroidota* species occupy a crucial ecological niche, acting as primary agents in the initial breakdown of recalcitrant organic carbon into bioavailable substrates [11]. One of the most important genera within this phylum is *Flavobacterium*, which is well-documented for its metabolic versatility and ability to break down a wide variety of organic materials, including complex carbohydrates and aromatic compounds. This genus plays a critical role in carbon cycling by facilitating the breakdown of plant polysaccharides and other organic matter in soil ecosystems [12,13].

Despite the ecological significance of *Flavobacterium*, new and previously unidentified species within this genus, particularly in specialized environments like black soils, remain poorly understood. Although several *Flavobacterium* species have been described from agricultural soils or rhizosphere-associated environments [14,15], these studies have mainly relied on cultured isolates, and genome-resolved evidence for uncultured *Flavobacterium* lineages in northeast black soils remains limited. As a result, it remains unclear whether black soils harbor phylogenetically distinct *Flavobacterium* populations with specific genomic adaptations to fluctuating carbon, nitrogen, and temperature regimes.

To address this gap, we used GMGA data to recover and characterize uncultured *Flavobacterium* MAGs from northeastern Chinese black soils. We successfully recovered three high-completeness MAGs from the black soil region of northeastern China, which represent a previously unknown evolutionary branch within the genus *Flavobacterium*. Phylogenomic analysis, supported by ANI and AAI comparisons, showed that these genomes (SRR24124297_bin.1, SRR24124298_bin.7, and SRR24124299_bin.22) represent a distinct uncultivated lineage. Because this lineage has not yet been isolated in pure culture, we followed the ICNP rules for its nomenclatural treatment [16,17]. Accordingly, we propose the Candidatus name *Candidatus Flavobacterium genomatis* based on genome-resolved metagenomic evidence.

## 2. Methods

### 2.1. Metagenomic Sequence Quality Control and Assembly

As part of the ongoing effort to build the GMGA, we analyzed publicly available metagenomic data. The environmental samples were collected from mixed soils in Jilin, China (43°60′ N 125°81′ E). All downloaded SRA files were first processed using the fastq-dump tool (v2.9.6) from sratoolkit v2.9.6 with the ‘-split-3′ parameter to separate paired-end raw reads. The raw reads were then quality-controlled using Trimmomatic v2.39 [18] to remove adapters and primers, and filter out low-quality reads with a length shorter than 50 bp or a quality score below 20. The remaining reads were assembled into single-sample contigs using MEGAHIT v1.2.9 [19], with a minimum contig length of 500 bp (-k-step 10 -k-min 27).

### 2.2. Metagenomic Binning and Refinement

Based on tetranucleotide frequency (TNF) and coverage information, MAGs were reconstructed using metaWRAP v1.2.2 [20] for this specific section of the GMGA project. Subsequently, the ‘bin_refinement’ function of metaWRAP v1.2.1 was used to refine the bins by integrating and improving the results from three different binning methods. During the refinement process, MAG completeness and contamination were assessed using CheckM2 v1.0.2 [21], and MAGs with completeness ≥ 50% and contamination <10% were selected. Ribosomal RNA (rRNA) genes, including 5S, 16S, and 23S subunits, were identified using Barrnap v0.9 [22], with the ‘-kingdom bac/arc’ parameter for bacteria. Transfer RNA (tRNA) genes were annotated using tRNAscan-SE v2.0.9 [23]. Based on the results of these analyses and the minimum information standards for metagenomic genomes (MIMAG) [24], high-completeness genomes were selected with >90% completeness, ≤5% contamination, ≥18/20 tRNA genes, and the presence of 5S, 16S, and 23S rRNA genes. The remaining genomes were categorized as medium quality.

### 2.3. Candidate Genome Identification and Reference Genome Collection

After binning, refinement, and genome quality assessment, all retained MAGs were taxonomically classified using GTDB v226 classification. This step was used solely for preliminary taxonomic screening to identify candidate MAGs affiliated with the genus *Flavobacterium*, rather than for final taxonomic assignment. MAGs assigned to *d_Bacteria, p_Bacteroidota*, *c_Flavobacteriia*, *o_Flavobacteriales*, *f_Flavobacteriaceae*, and *g_Flavobacterium*, but lacking a confident species-level annotation (*s_*), were retained as candidate genomes for downstream taxonomic evaluation. This screening yielded three candidate MAGs: SRR24124297_bin.1, SRR24124298_bin.7, and SRR24124299_bin.22. To determine their phylogenetic placement and taxonomic status, we collected genome data for 812 validly published species within the genus *Flavobacterium* from public databases, with priority given to type-strain genomes where available. Reference genomes with completeness > 90% and contamination < 5% were preferentially retained. However, for species lacking a genome that fully satisfied these criteria, the highest-quality available representative genome was retained to preserve species-level coverage and ensure the overall completeness and stability of the phylogenetic framework. Accession numbers, GTDB classifications, and CheckM2-based completeness and contamination values for all reference genomes used in the tree are provided in Table S1. In addition, one representative genome from the genus *Flavivirga* was selected as the outgroup. The final dataset used for phylogenomic analysis therefore consisted of the three candidate MAGs, 812 reference *Flavobacterium* genomes, and one outgroup genome.

### 2.4. Phylogenomic Analysis

First, a multiple sequence alignment (MSA) was generated based on 120 bacterial single-copy marker genes identified and aligned using GTDB-Tk v2.3.2 [25]. A maximum likelihood phylogenetic tree was constructed using IQ-TREE v2.0 [26]. The phylogenetic model selection was automatically performed by ModelFinder, with the best-fitting model being TIMe + R7, which was consistently supported by the Akaike Information Criterion (AIC), corrected AIC (c-AIC), and Bayesian Information Criterion (BIC). The quality-controlled alignment results were then re-analyzed in IQ-TREE to build maximum likelihood phylogenetic trees based on marker proteins and ribosomal proteins, respectively. Node support and tree robustness were assessed using the ultrafast bootstrap method with 1000 replicates (parameters: ‘-alrt 1000 -bb 1000 -nt AUTO -m MFP’). Genome-relatedness indices were calculated to evaluate the taxonomic position. The ANI value was calculated using FastANI 1.33 [27], and this was compared with the recommended 95% threshold for species delineation. Additionally, the AAI profile was computed using EzAAI 1.2.1 [28]. Prior to these pairwise calculations, open reading frames were predicted using Prodigal v2.6.3. The resulting AAI values were compared with the commonly recommended 65% threshold for genus delineation [29].

### 2.5. Gene Functional Annotation

Initial functional annotation was performed using eggNOG-mapper v2 [30]. The annotation file was generated by comparing the results with the KEGG (Kyoto Encyclopedia of Genes and Genomes) database [31] using the parse_eggNOG.py script, and functional information was retrieved from the KEGG database using the ko00001.json (25 September 2025). This mapping was used to reconstruct metabolic pathways and evaluate their completeness.

### 2.6. Global Abundance Analysis

To evaluate the ecological distribution of the candidate lineage on a global scale, we queried and analyzed public environmental metagenomic data from the MicrobeAtlas project (https://microbeatlas.org/ (accessed on 4 March 2026)) [32]. MicrobeAtlas is an online database that integrates and standardizes large-scale public 16S rRNA data, encompassing environmental microbiome samples from diverse ecosystems worldwide. It performs hierarchical OTU clustering based on full-length 16S rRNA sequence similarity thresholds (99%), which typically corresponds to species-level taxonomic units. In this study, we selected a high-quality representative genome (SRR24124298_bin.7) containing a complete 16S rRNA gene, extracted its 16S rRNA gene sequence, and used it as a query in the MicrobeAtlas database. To assess whether additional 16S rRNA gene copies were present in the representative genome, the recovered 16S rRNA sequence from SRR24124298_bin.7 was used as a BLASTn v2.13.0+ query against both the assembled MAG and the corresponding sample-level contig assembly, retaining only hits with ≥90% identity and ≥90% query coverage. The retrieved occurrence records were then analyzed across global environmental samples. Based on the obtained standardized occurrence and abundance data, we integrated the geographic location information of the samples and further classified them by environmental source into aquatic, soil, plant, and animal habitats to assess the global distribution pattern and relative abundance of the candidate lineage in different ecosystems.

### 2.7. Statistical Analysis

All statistical analyses for figure generation were conducted in R environment v4.1.2 [33]. The results were visualized using R packages tidyverse v2.0.0, reshape2 v1.4.4, dplyr v1.1.4, and ggplot2 v4.0.0 [34,35].

## 3. Results and Discussion

### 3.1. Genomic Characteristics and Quality Assessment

From the candidate MAGs preliminarily assigned to the genus *Flavobacterium*, three genomes with high completeness (>90%) and low contamination (<5%) were retained for downstream analyses [24]. Three MAGs were identified: SRR24124297_bin.1 (completeness 96.74%, contamination 0.11%), SRR24124298_bin.7 (completeness 99.99%, contamination 1.67%), and SRR24124299_bin.22 (completeness 99.95%, contamination 0.49%) (see Table 1). These genomes range in size from 2.7 to 2.9 Mb and show a highly conserved G + C content of approximately 48%. Additionally, these genomes display notable similarity in terms of coding density and gene length. These features reflect significant genetic diversity and adaptability within this unclassified group of the genus, further supporting their potential as a new species. From the candidate MAGs preliminarily assigned to the genus *Flavobacterium*, three genomes with high completeness (>90%) and low contamination (<5%) were retained for downstream analyses [24]. SRR24124297_bin.1 and SRR24124299_bin.22 were classified as medium-quality MAGs due to incomplete rRNA recovery (Table 1). Because the three genomes share very high pairwise ANI values (99.88–99.97%) and show concordant phylogenomic placement, SRR24124297_bin.1 and SRR24124299_bin.22 were regarded as non-type conspecific genomes and were retained as supportive evidence for comparative genomic analysis [27].

### 3.2. Phylogenetic Analysis of MAGs

**Figure 1:**
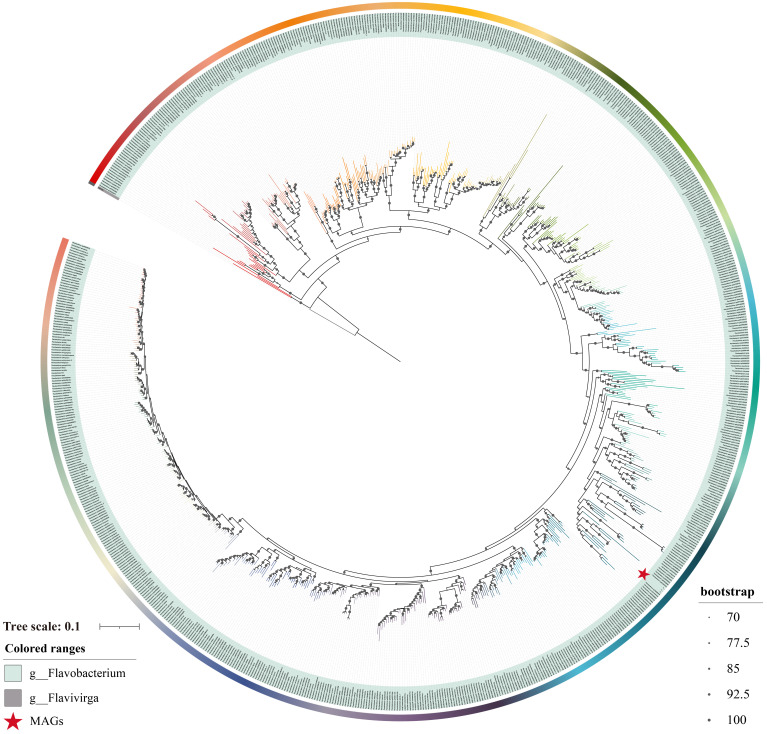
Maximum-likelihood phylogenomic tree based on 120 bacterial marker genes. *Flavivirga* was used as the outgroup to root the tree. Bootstrap values greater than 70% are shown at the nodes. A total of 812 species within the genus were included, and the three genomes recovered in this study are indicated by red stars. The scale bar represents one substitution per site. The external colored circle indicates species-level taxonomic identities within the genus *Flavobacterium*. Because most reference taxa represent different species, the color differences are intended to provide an overview of species-level diversity rather than to distinguish each species individually in the main figure. The scale bar represents one substitution per site.

To determine their phylogenetic position, we performed a phylogenetic analysis using 812 validly published *Flavobacterium* species listed in LPSN (List of Prokaryotic names with standing in nomenclature). The corresponding genomes were retrieved from public databases, with priority given to type strain genomes. The genome of *Flavivirga* sp., a member of the family *Flavobacteriaceae* within the order *Flavobacteriales*, was included as the outgroup. Phylogenetic analysis based on 120 single-copy marker genes showed that these three unclassified MAGs formed a distinct monophyletic group within the genus *Flavobacterium*, with 100% bootstrap support, indicating their unique evolutionary position within the genus (Figure 1).

### 3.3. Classification Supported by ANI and AAI Values

**Figure 2:**
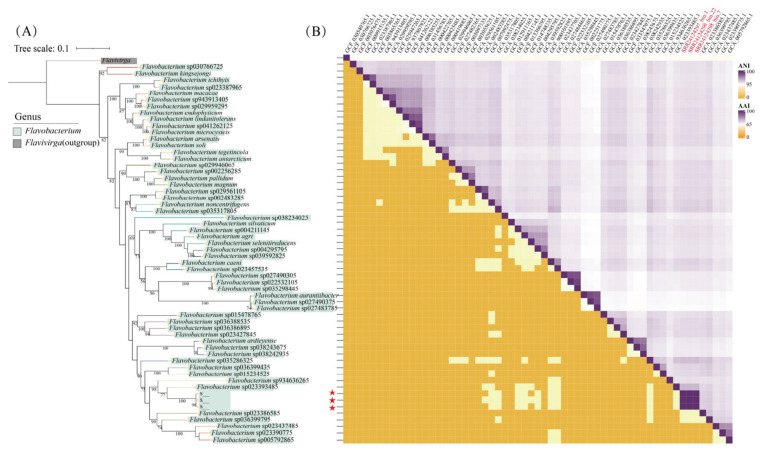
Local phylogenomic placement and pairwise genomic relatedness of the three recovered genomes. (**A**) Local maximum-likelihood phylogenetic tree showing the branch containing the three genomes recovered in this study within the genus *Flavobacterium*. *Flavivirga* was used as the outgroup to root the tree. Bootstrap values greater than 70% are indicated at the nodes. (**B**) Composite heatmap of pairwise ANI and AAI values among the selected genomes, used to evaluate genomic relatedness and taxonomic placement. White indicates the commonly used species-level threshold for ANI (95%). AAI values are shown for comparative context and were interpreted together with phylogenomic relationships, rather than using a fixed universal genus cutoff. The 65% label on the AAI color scale is retained only as a visual reference and was not used here as a strict universal cutoff for genus delineation. Red stars indicate the three MAGs recovered in this study, namely SRR24124297_bin.1, SRR24124298_bin.7, and SRR24124299_bin.22, which support the proposal of *Candidatus Flavobacterium genomatis*.

We further assessed the taxonomic placement of the three MAGs using ANI and AAI. The pairwise ANI values among the three MAGs exceeded the species delineation threshold of 95% (99.88–99.97%), whereas ANI values between the MAGs and reference genomes were all below 95%, strongly supporting their classification as a novel species (Figure 2; Table S2). In addition, pairwise AAI values among the three MAGs were 99.95–99.97%, while AAI values between the MAGs and the selected reference genomes ranged from 62.93% to 76.05% (Figure 2; Table S3). Because genus-level AAI boundaries are lineage-dependent and current genome-based taxonomic standards treat AAI as a guiding comparative metric rather than a universally fixed cutoff, these values were interpreted together with phylogenomic placement and reference-genome context, rather than against a single rigid threshold [36,37]. This interpretation is particularly important for *Flavobacterium*, for which additional in-depth phylogenetic and phylogenomic analyses are often required for robust taxonomic designation [38]. Under this integrated framework, the three MAGs are consistently placed within the genus *Flavobacterium* and represent a distinct novel species within this genus.

### 3.4. Taxonomic Placement and Nomenclatural Considerations

Among the three recovered MAGs, SRR24124298_bin.7 is proposed as the type strain for the new species *Candidatus Flavobacterium genomatis.* This specific genome meets the high-quality MIMAG standards [24] and retains both 16S and 23S rRNA genes. The classification of its 16S rRNA gene is consistent with that of the whole genome (Table 2). The other two MAGs, SRR24124297_bin.1 and SRR24124299_bin.22, are not treated as type material. Rather, they are considered non-type conspecific genomes because they share 99.88–99.97% ANI with SRR24124298_bin.7 and cluster with it consistently in the phylogenomic analyses. These additional genomes therefore provide supportive evidence for species circumscription and comparative genomic coherence. This lineage was named under the ICNP nomenclatural framework. The genome sequence of SRR24124298_bin.7 has been deposited in a public genome repository and is used here as genomic evidence supporting the proposal of *Candidatus Flavobacterium genomatis* [39].

### 3.5. Metabolic Potential of Candidatus Flavobacterium genomatis

Based on metabolic annotation analyses, this novel taxon possesses a complete central carbon metabolic hub, including glycolysis/gluconeogenesis pathways and the tricarboxylic acid (TCA) cycle. Its central carbon metabolism network exhibits high integration and plasticity, likely contributing to its adaptation to complex soil environments [40]. The species also has active starch and sucrose metabolism, with its genome encoding key enzymes such as amylase (*AMY*), maltose-utilizing enzyme (*mapA*), and phosphoglucomutase (*pgmB*), enabling efficient conversion of insoluble starch and maltose into D-glucose, which is directly fed into glycolysis. The presence of phosphoenolpyruvate carboxylase (*Ppc*) suggests a capacity for anaplerotic CO_2_ sequestration, which serves to replenish key TCA cycle intermediates, such as oxaloacetate. This metabolic strategy likely supports cellular homeostasis during carbon source fluctuations or periods of high biosynthetic demand [41]. This compensatory mechanism stabilizes cycle flux and ensures continuous energy and precursor supply [42].

In terms of nutrient acquisition and metabolic homeostasis, the species’ genome encodes a diverse and specialized ABC transporter system. It includes a complete iron complex uptake system, which facilitates the efficient acquisition of this essential trace element in black soils, where iron ions are often chelated by humic substances. The genome also encodes a copper ion processing system, likely associated with its respiratory chain. Moreover, the metabolic profile reveals specific transport modules involved in phospholipid turnover, lipopolysaccharide assembly, lipoprotein secretion, and cell division, forming a comprehensive network for material transport and cellular homeostasis.

The taxon has also evolved a streamlined and efficient stress response mechanism to cope with environmental changes. Cold shock protein *CspA* is detected in the genome, acting as an RNA chaperone to reduce mRNA secondary structure inhibition of translation under low-temperature stress, ensuring efficient protein synthesis. In addition, its encoded catalase-peroxidase (*KatG*) removes H_2_O_2_ and organic peroxides, mitigating oxidative damage. Combined with molecular chaperones and protein degradation factors, this system likely plays a key role in maintaining cellular homeostasis under low temperature and oxidative stress [43].

Furthermore, the metabolic characteristic of this lineage is deeply involved in the nitrogen biogeochemical cycle in soil environments, with high adaptability to agricultural nitrogen input [44]. The metabolic map clearly shows its active nitrogen metabolism potential, including ammonia assimilation via glutamine synthetase (*glnA*) and partial denitrification processes, driven by nitrite reductase (*nirBD*) and nitric oxide reductase (*norB*) [45]. Notably, the genome encodes nitrous oxide reductase (NosZ), which catalyzes the terminal step of denitrification by reducing N_2_O to N_2_ [46]. The presence of this gene suggests that this lineage has the genomic potential to complete the final step of denitrification and may therefore contribute to N_2_O reduction under suitable environmental conditions. However, because our evidence is based on genome annotation alone, without metatranscriptomic, isotopic, cultivation-based, or direct process-level measurements, we do not infer confirmed in situ N_2_O reduction activity or classify this organism as a demonstrated greenhouse gas sink [47].

**Figure 3:**
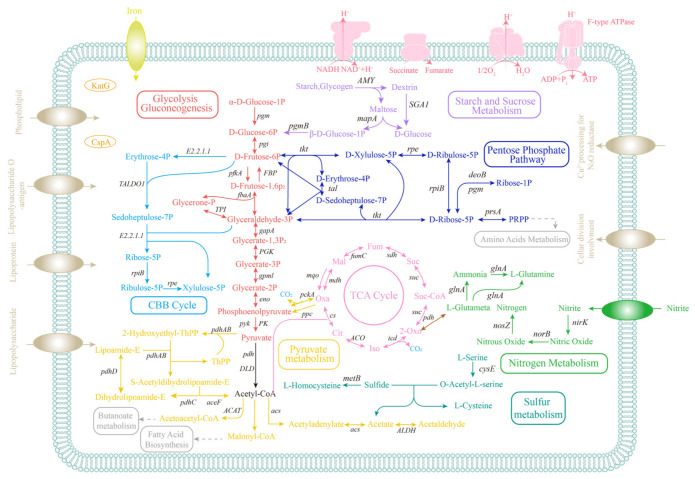
Overview of the predicted metabolic potential of *Candidatus Flavobacterium genomatis*. This metabolic map was reconstructed from genome annotations of the three recovered MAGs and represents inferred metabolic capabilities rather than experimentally validated in situ activities. This metabolic map was reconstructed based on genome annotation of the three recovered MAGs. The diagram summarizes the major metabolic modules identified in this lineage, including central carbon metabolism, starch and sucrose metabolism, nitrogen metabolism, sulfur metabolism, fatty acid biosynthesis, and oxidative phosphorylation. Membrane-associated transporters and respiratory complexes are shown along the cellular envelope. Different colors are used only to distinguish metabolic modules, transport processes, and graphical elements; they do not represent quantitative differences in pathway activity, gene abundance, or experimentally measured metabolic fluxes. Arrows indicate predicted biochemical reactions or transport directions based on genome annotation. This figure was generated according to the gene annotation data using Adobe Illustrator 2023 software.

In response to the unique physical conditions of the northeast, the species exhibits significant respiratory metabolic flexibility. Its genome retains various membrane-associated components of the electron transport chain, including NADH dehydrogenase, terminal oxidases, and F-type ATPase, suggesting it can couple reducing power generated from central metabolism to membrane potential establishment and ATP synthesis. The sulfur metabolism pathway, involving cysteine synthesis by *cysE* and *cysK*, assimilates inorganic sulfur and directs it towards the synthesis of sulfur-containing amino acids, providing essential precursors for protein synthesis and reductive metabolism under low-temperature and oxidative stress [48]. These features collectively support the successful colonization of this novel *Flavobacterium* species in the dynamic black soil ecosystem. (Figure 3).

### 3.6. Global Biogeography and Ecological Niche

**Figure 4:**
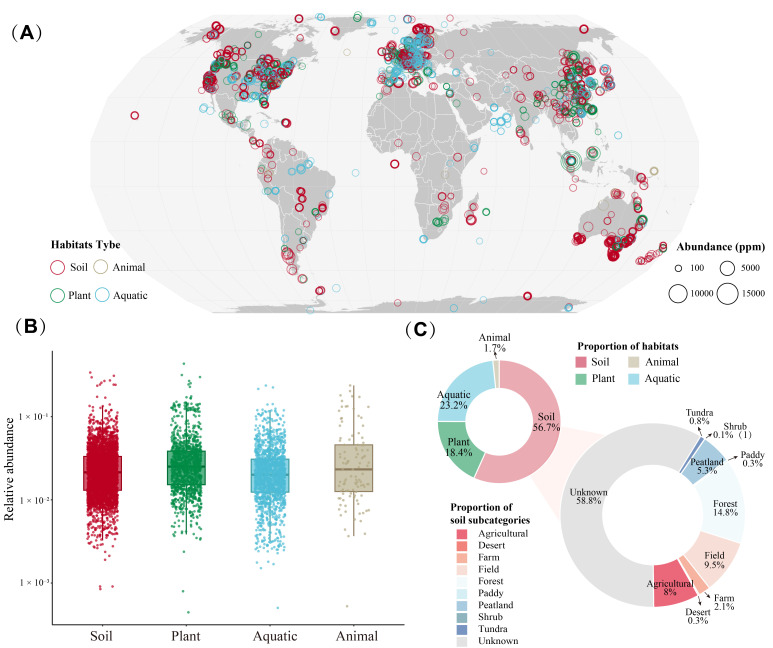
Global distribution and habitat composition of *Candidatus Flavobacterium genomatis* based on 16S rRNA gene sequence mining. (**A**) Global distribution of detected sequences. Open circles indicate metagenomic sampling sites. Circle size is proportional to relative abundance, and border color denotes the corresponding habitat category. (**B**) Distribution of relative abundance values across different habitat types. The y-axis is shown on a log10 scale. (**C**) Habitat composition of the detected occurrences of *Candidatus Flavobacterium genomatis* across the four major habitat categories. This panel summarizes the relative distribution within positive records only; statistical enrichment relative to the full MicrobeAtlas background was evaluated separately using one-sided Fisher’s exact tests with Benjamini–Hochberg correction.

We queried the MicrobeAtlas database using the 16S rRNA gene sequence of *Candidatus Flavobacterium genomatis* and detected closely related records (≥99.7% sequence similarity) across diverse environments, including soil, aquatic, plant-associated, and animal-associated habitats. Prior BLASTn validation against both the assembled MAG and the corresponding contig assembly recovered only a single full-length 16S rRNA hit, with no additional 16S-like loci detected, supporting the use of this sequence as the representative marker for database-based distribution analysis. Among the positive records, soil-associated samples accounted for the largest proportion (56.7%), followed by aquatic (23.2%), plant-associated (18.4%), and animal-associated samples (1.7%). To account for habitat representation bias in the MicrobeAtlas database, we further compared habitat composition in the positive records with background habitat representation in the full database using one-sided Fisher’s exact tests with Benjamini–Hochberg correction. Soil-associated samples remained significantly enriched after this correction (56.7% in positive records vs. 16.8% in the background; odds ratio = 6.48, FDR < 0.001), supporting a non-random association with soil environments. Aquatic and plant-associated samples also showed enrichment trends, whereas no enrichment was detected for animal-associated samples (Table S6). Among soil-associated records, agricultural soils were most common, followed by forests and peatlands, while tundra and desert soils showed minimal representation. The relatively frequent occurrence of this lineage in temperate, organic matter-rich soils suggests adaptation to such environments, and northeast black soils may represent a favorable habitat for this lineage. Overall, these distribution patterns are consistent with the genomic features of *Candidatus Flavobacterium genomatis*, particularly its central carbon metabolism and polysaccharide degradation potential. (Figure 4).

## 4. Conclusions

This study characterized three MAGs from soil samples in the northeast black soil region of China, representing a novel lineage within the genus *Flavobacterium*. We propose *Candidatus Flavobacterium genomatis* based on the metagenomic evidence and ICNP nomenclatural rules. Functional annotation reveals efficient plant polysaccharide degradation and a chemoheterotrophic lifestyle, along with proteins involved in low-temperature and oxidative stress responses. The presence of genes associated with denitrification, particularly nitrous oxide reductase, suggests that this lineage may contribute to nitrogen transformation and potential N_2_O reduction under suitable environmental conditions. These metabolic flexibilities, along with stress response capabilities, support its successful colonization and ecological function in carbon-rich, high-nitrogen input black soil environments. Analysis of 6454 samples based on 16S rRNA gene sequence reveals a clear global distribution with pronounced habitat specificity, exhibiting the highest detection frequency and relative abundance in soil habitats, particularly in agricultural soils. The northeast black soil region of China, characterized by low temperatures, seasonal freeze–thaw cycles, and rich organic inputs, is identified as a key distribution hotspot. This finding expands the diversity of *Flavobacterium* and provides new insights into uncultured terrestrial microorganisms. Because the present study is based on only three conspecific MAGs, the functional and ecological patterns described here should be interpreted as initial genome-based characteristics of the currently recovered representatives, rather than as exhaustive features of the taxon across all environments.

Description of *Candidatus Flavobacterium genomatis*. *Candidatus Flavobacterium genomatis* (ge.no.ma’tis. N.L. gen. n. *genomatis*, of a genome). The description is the same as for the genus. The taxon is represented by the metagenome-assembled genome SRR24124298_bin.7. This lineage has not yet been isolated in pure culture, and the genome was assembled from metagenomic data derived from a black soil sample collected in Jilin, China.

## Figures and Tables

**Table 1:** General features of the *Candidatus Flavobacterium genomatis*.

MAGs	SRR24124297_bin.1	SRR24124298_bin.7	SRR24124299_bin.22
Completeness (%)	96.74	99.99	99.95
Contamination (%)	0.11	1.67	0.49
Genome_Size	2,789,768	2,907,874	2,743,091
Coding_Density	0.915	0.913	0.916
Contig_N50	48,925	189,887	191,408
Average_Gene_Length (bp) ^a^	338.86	344.33	345.78
Total_Coding_Sequences	2516	2575	2426
GC_Content	48%	48%	48%
No. of 16S rRNA genes ^b^	0	1	0
No. of 23S rRNA genes ^b^	0	3	0
No. of 5S rRNA genes ^b^	1	3	2
tRNAs ^c^	21	22	21
MIMAG Quality ^d^	Medium	High	Medium
Source Run ID	SRR24124297	SRR24124298	SRR24124299

^a^ Estimated by tripling the mean amino acid sequence length of the annotated proteins. ^b^ Barrnap (version 0.9) was applied using the bacterial-specific database (-k bac) to evaluate the copy number of 16S, 23S and 5S rRNA genes. The reported figures encompass both intact and fragmented gene sequences. ^c^ The total count of transfer RNAs was computed via tRNAscan-SE (v2.0). A minimum threshold of 18 tRNAs is generally expected to meet the high-quality MAG criteria. ^d^ Quality tiers are defined following the MIMAG framework [24]. The “High-quality” designation requires completeness exceeding 90%, contamination below 5%, the detection of all three rRNA genes (5S, 16S, and 23S), and at least 18 tRNAs. “Medium-quality” designates MAGs with >50% completeness and <10% contamination.

**Table 2:** Description of *Candidatus Flavobacterium genomatis*.

Item	Description
Nomenclatural treatment	Candidatus taxon proposed for an uncultivated prokaryote under an ICNP/ICSP-aligned nomenclatural framework
Proposed Candidatus name	*Candidatus Flavobacterium genomatis*
Specific epithet	genomatis
Etymology	ge.no.ma’tis. N.L. gen. n. *genomatis*, of a genome.
Cultivation status	Not isolated in pure culture
Basis of proposal	Genome-resolved metagenomic evidence from black soil metagenomes
Representative genome	SRR24124298_bin.7
Representative genome accession	GWHHOSV00000000.1
Genome status	Incomplete (High-quality MAG)
Genome size	2,907,874 bp
GC mol%	48.0
16S rRNA gene source	SRR24124298
Country of origin	China
Description of the new taxon and diagnostic traits	This novel bacterial taxon, identified via a metagenome-assembled genome recovered from soil, belongs to the genus *Flavobacterium*. The taxon is represented by SRR24124298_bin.7. These MAGs exhibit high genomic coherence (ANI ≥ 99.88 %) but significant divergence from all other known species in the genus. Phylogenomic analyses indicate that it forms a distinct lineage within the genus, with clear genomic divergence from all currently described species. Functional genomic analysis reveals that this species possesses an efficient plant polysaccharide degradation capability and a chemoheterotrophic metabolic mode, along with encoded stress-response proteins. The detection of a complete denitrification pathway suggests its potential dual ecological roles in organic matter transformation and greenhouse gas mitigation within nitrogen-enriched black soil ecosystems. These genomic features suggest adaptations to nutrient-variable soil environments, distinguishing this taxon from closely related species. Screening of public databases shows this lineage is widespread globally, primarily living in diverse soil.
Region of origin	Jilin province
Source	Soil
Sampling date	April 2021
Latitude & Longitude	43°60′ N 125°81′ E

## Data Availability

The five novel MAGs described in this study were generated as part of our group’s Global Mollisols Genomic Atlas (GMGA) project. These genomes have been deposited in the National Genomics Data Center (NGDC) Genome Warehouse (GWH) under BioProject PRJCA057914, with specific accession numbers GWHHOSW00000000.1, GWHHOSV00000000.1, and GWHHOSU00000000.1. The original raw metagenomic datasets used to build this atlas subset are publicly available in the NCBI Sequence Read Archive (SRA) under BioProject PRJNA953804 (runs SRR24124297, SRR24124298, and SRR24124299). The representative genome supporting the proposal of *Candidatus Flavobacterium genomatis* is SRR24124298_bin.7, corresponding to accession number GWHHOSV00000000.1. Custom bioinformatic scripts are accessible via https://github.com/xiaoyuezhang289-arch/blacksoil_newspecies (accessed on 23 March 2026).

## Supplementary Materials

The following supporting information can be downloaded at https://www.mdpi.com/article/10.3390/microorganisms14061292/s1. Table S1. CheckM2-based genome quality assessment and GTDB classification; Table S2. Pairwise Average Nucleotide Identity (ANI) values among the genomes; Table S3. Pairwise Average Amino Acid Identity (AAI) matrix among the genomes; Table S4. Ecological habitat distribution, source characteristics, and abundance of the target taxon with sequencing metadata across samples; Table S5. KEGG Orthology (KO) distribution matrix of the compared genomes; Table S6. Enrichment analysis of major habitat categories for *Candidatus Flavobacterium genomatis* in the MicrobeAtlas database; Table S7. BLASTn verification of the recovered 16S rRNA locus in the type genome SRR24124298_bin.7 and its corresponding contig assembly. Supplementary Code contains cluster-based scripts, R scripts, optional intermediate scripts, and a README file for reproducing the main analyses and figures.
